# Supplementary material for: Analysis of Metabolic Components of JUNCAO Wine Based on GC-QTOF-MS
Source: Foods. 2023 Jun 3;12(11):2254. doi: 10.3390/foods12112254 (PMC10252805; doi:10.3390/foods12112254)
Supplement: Supplementary file 1 [file foods-12-02254-s001.zip › foods-2354671-supplementary/foods-2354671-supplementary-sentconversion/Supplementary File/supplementary figures.pdf]

Total ion chromatogram of metabolic components in different fermentation analyzed by GC-QTOF-MS

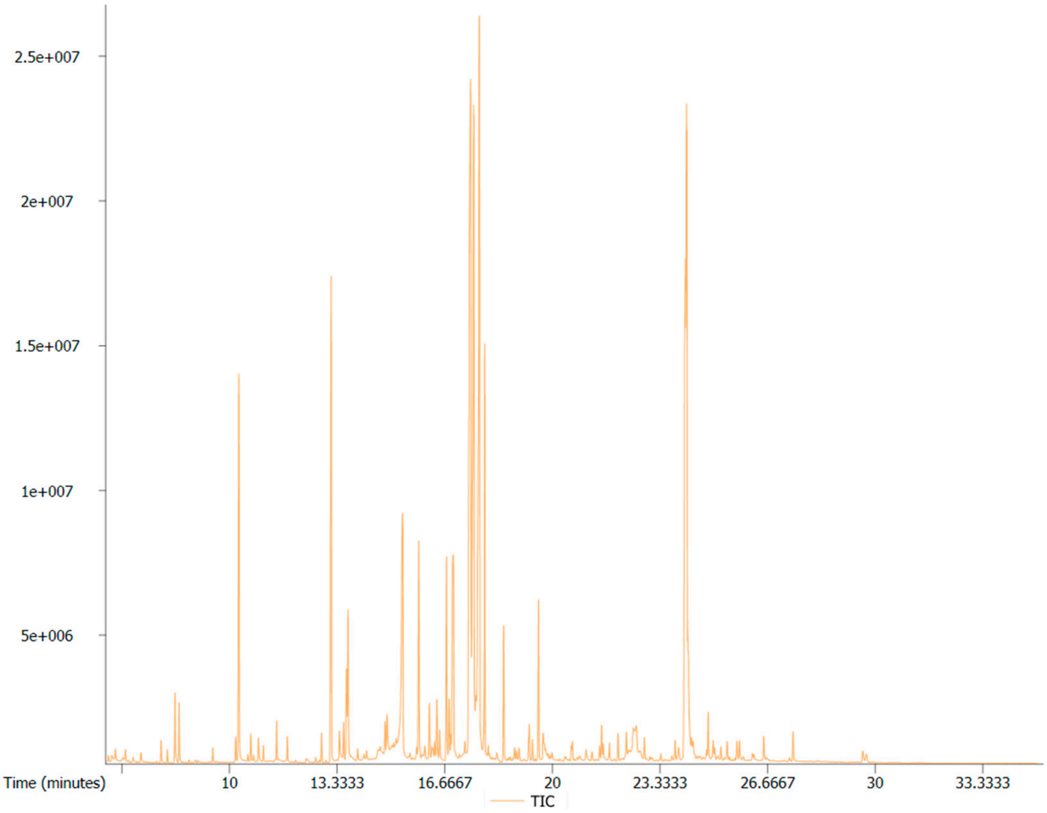

Figure S1. Total ion chromatogram of QC sample on day 0

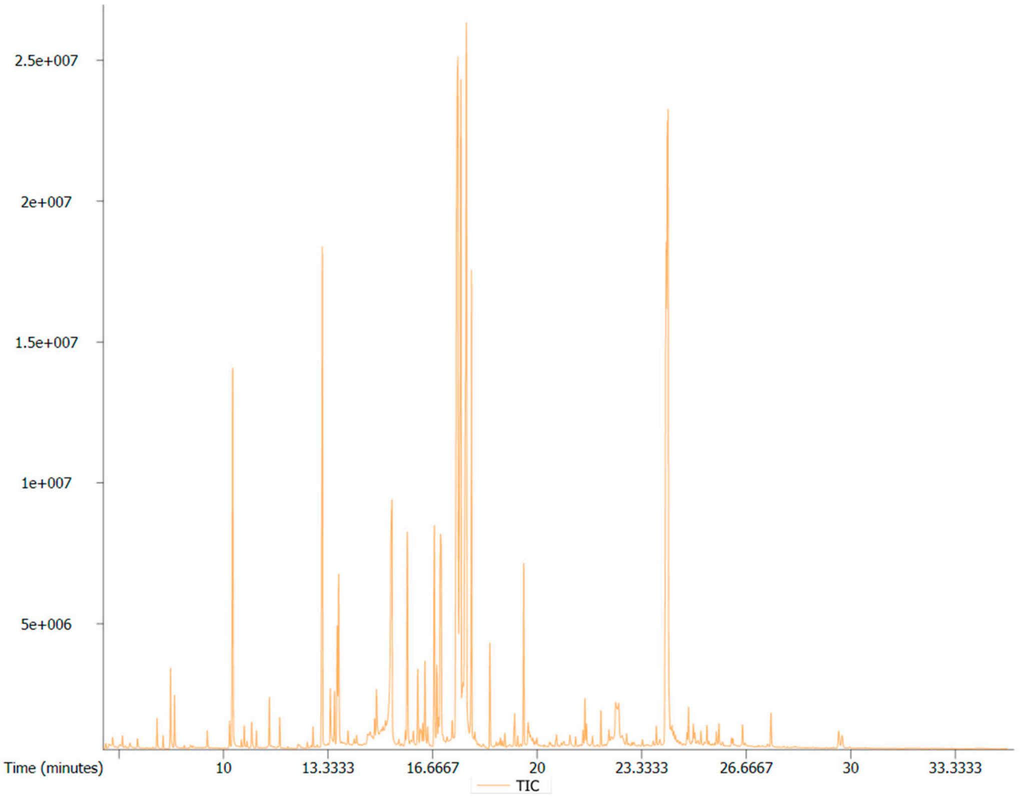

Figure S2. Total ion chromatogram of QC sample on day 0

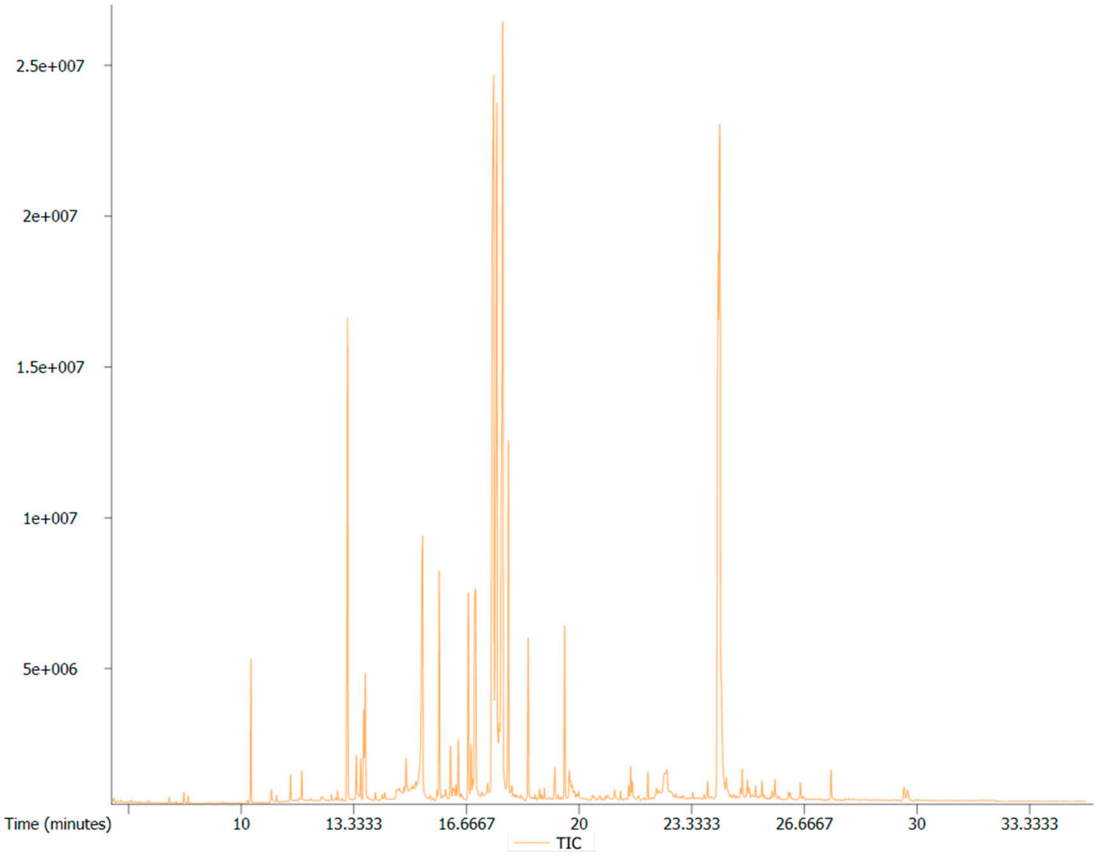

Figure S3. Total ion chromatogram of QC sample on day 0

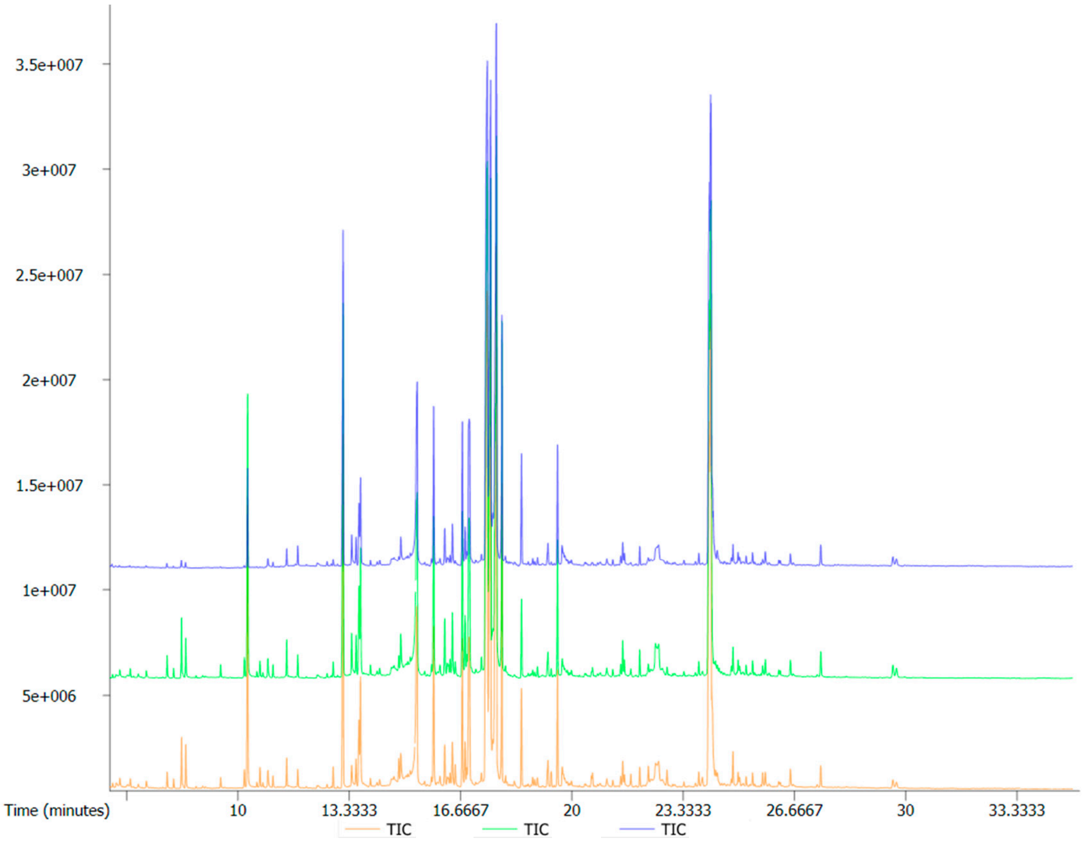

Figure S4. Total ion chromatogram of superimposed QC samples on day 0

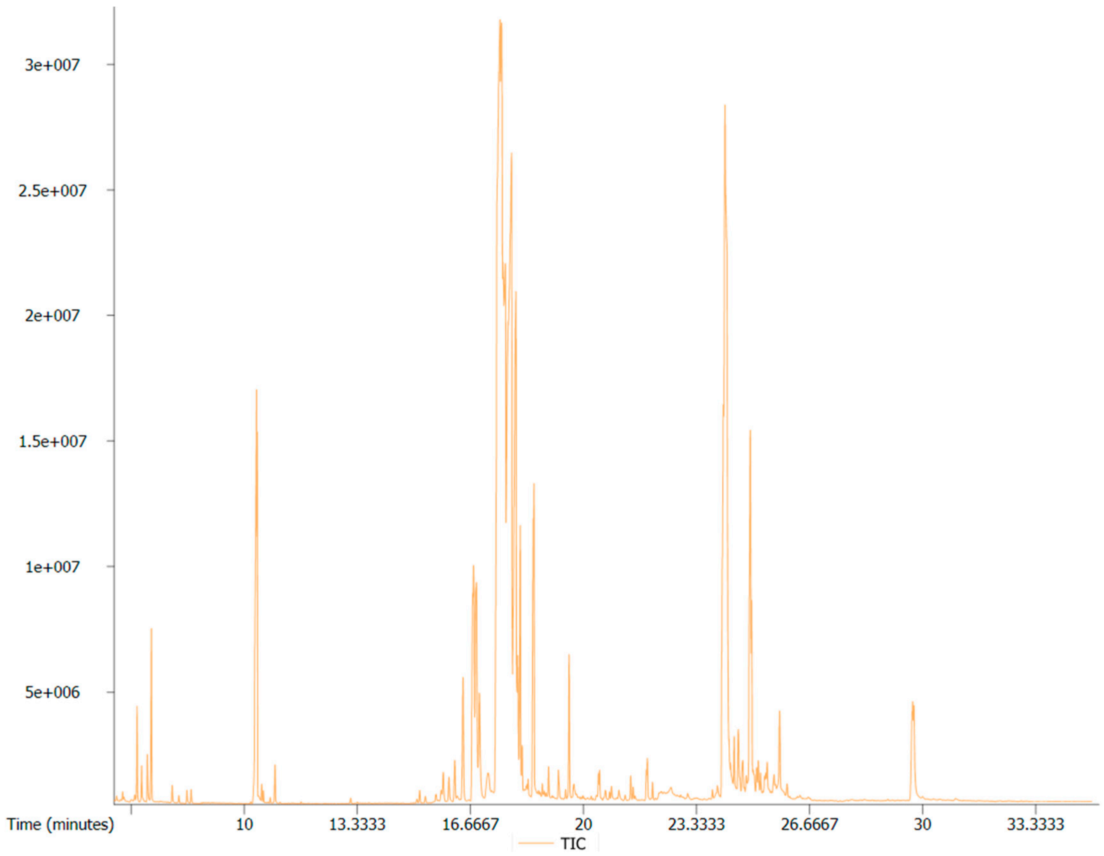

Figure S5. Total ion chromatogram of QC sample on day 2

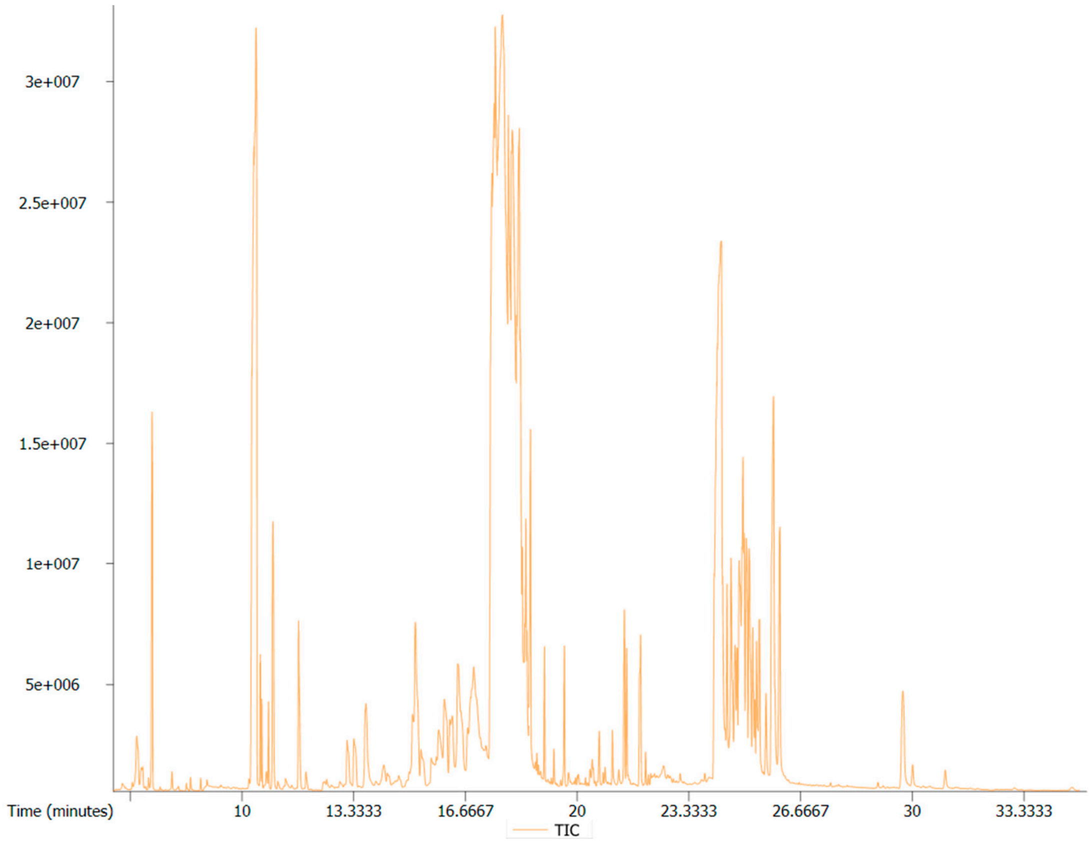

Figure S6. Total ion chromatogram of QC sample on day 2

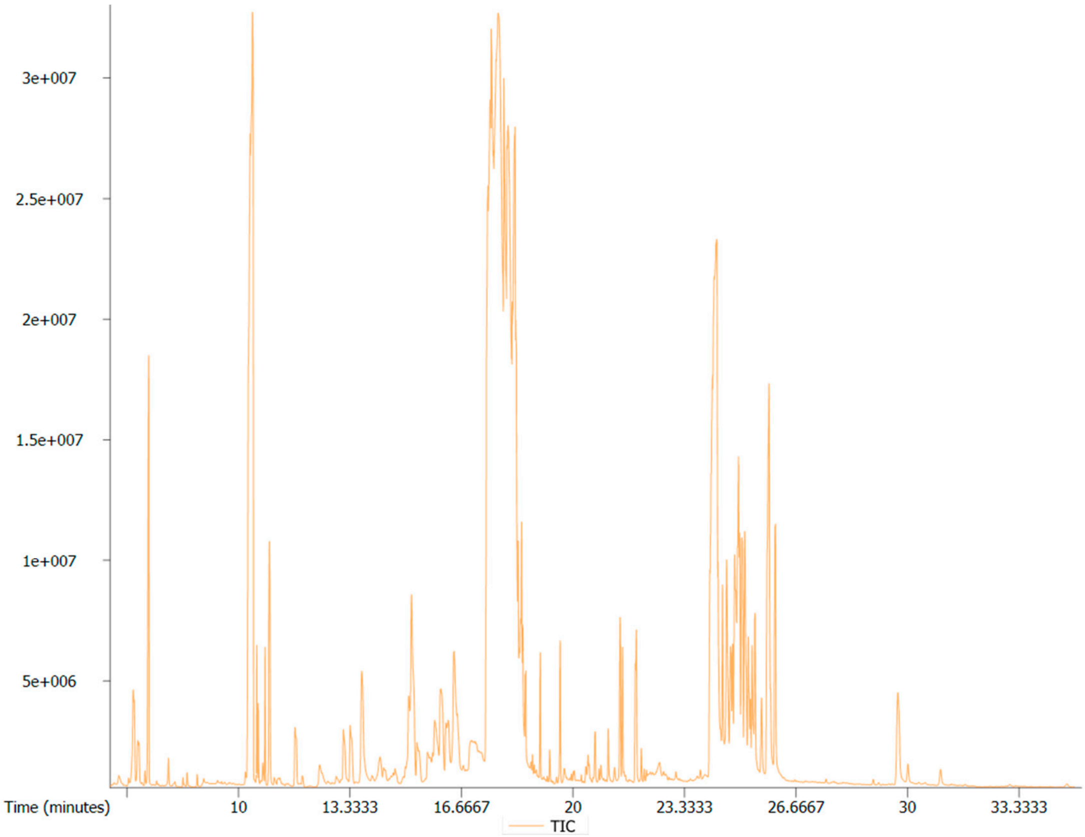

Figure S7. Total ion chromatogram of QC sample on day 2

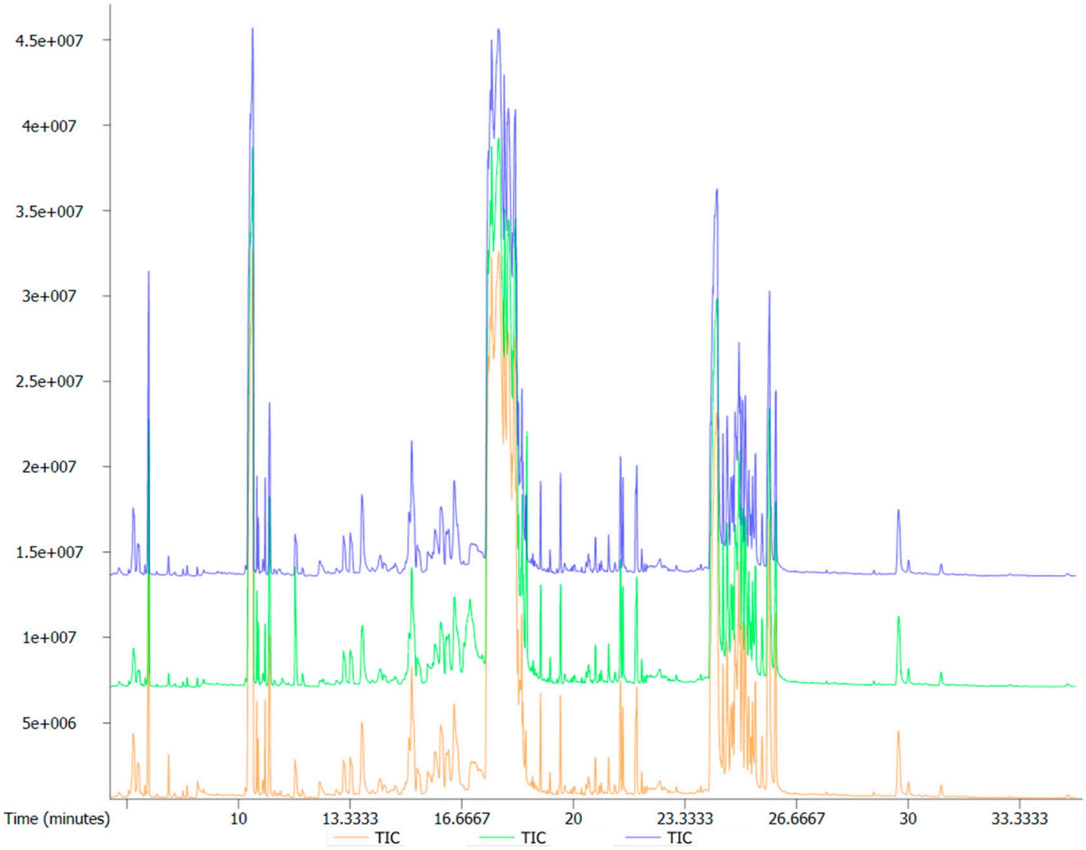

Figure S8. Total ion chromatogram of superimposed QC samples on day 2

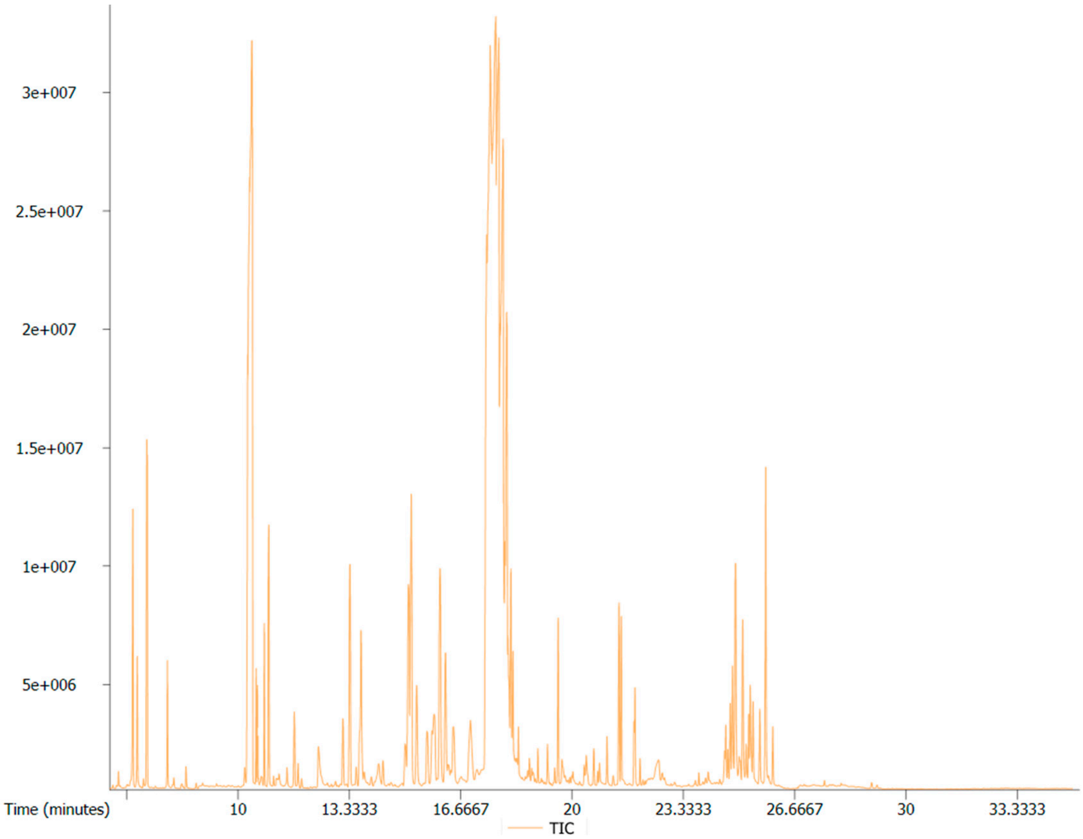

Figure S9. Total ion chromatogram of QC sample on day 10

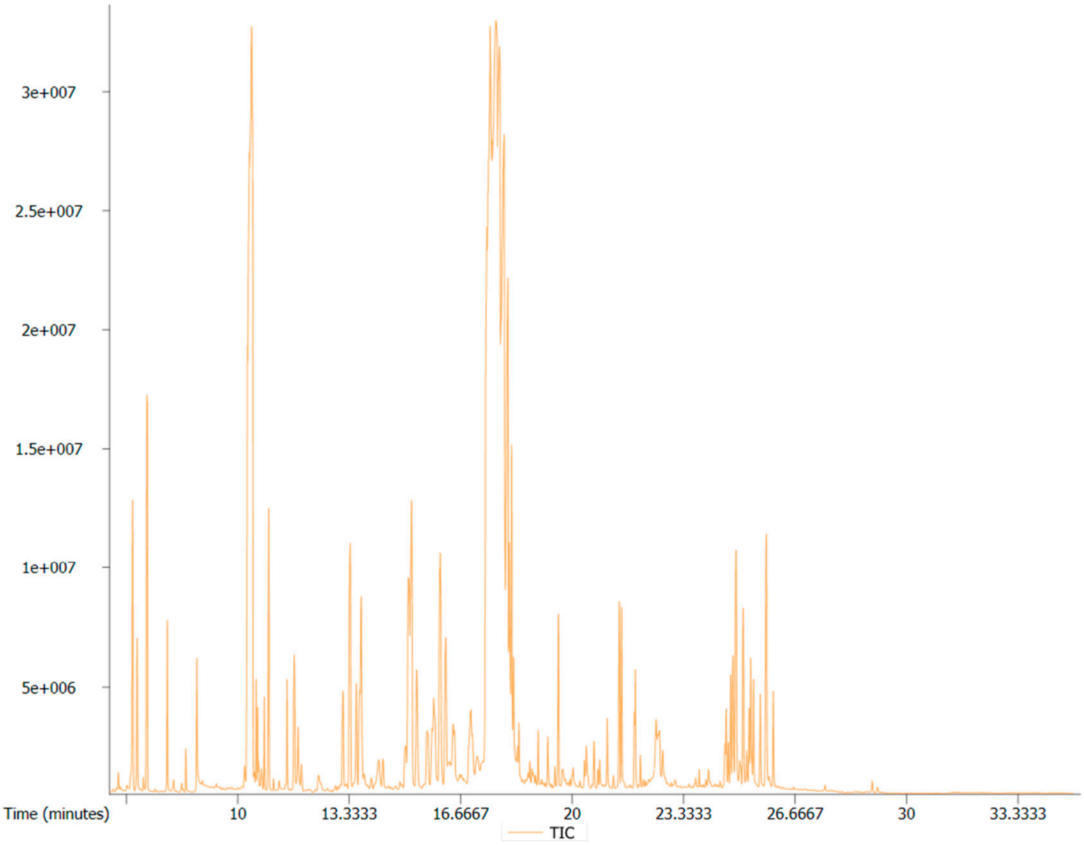

Figure S10. Total ion chromatogram of QC sample on day 10

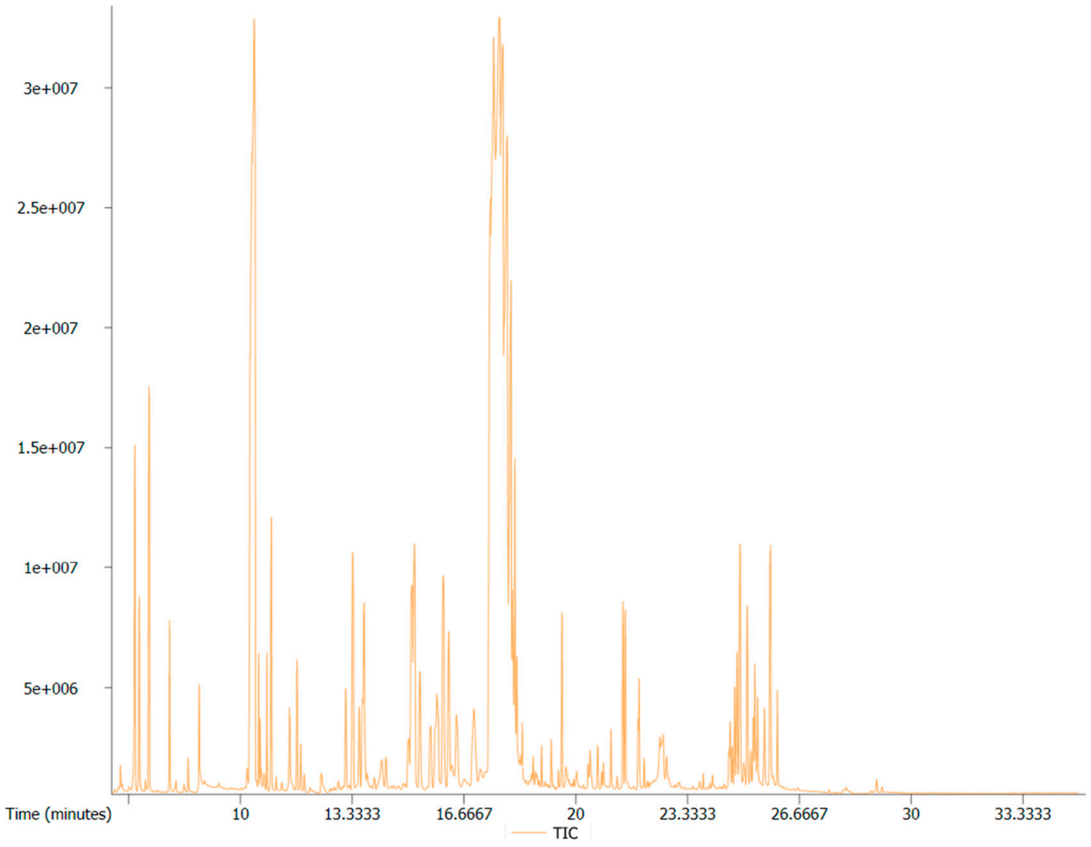

Figure S11. Total ion chromatogram of QC sample on day 10

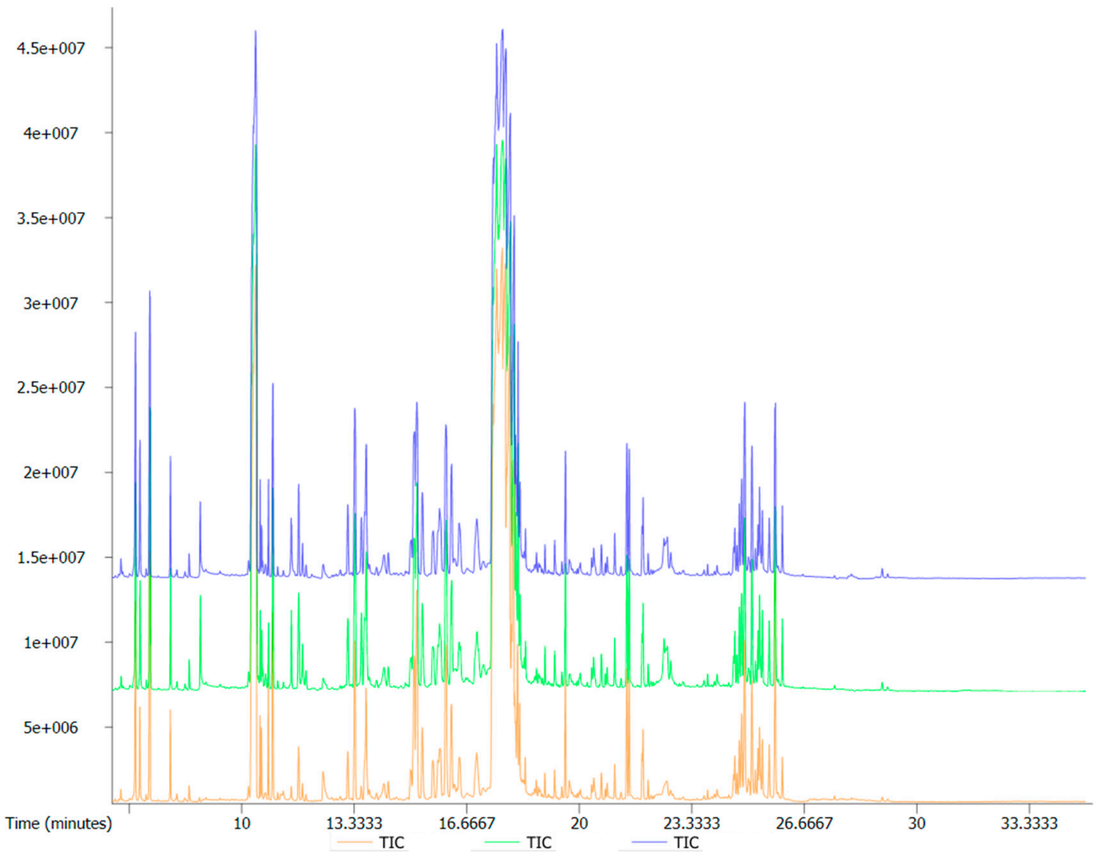

Figure S12. Total ion chromatogram of superimposed QC samples on day 10

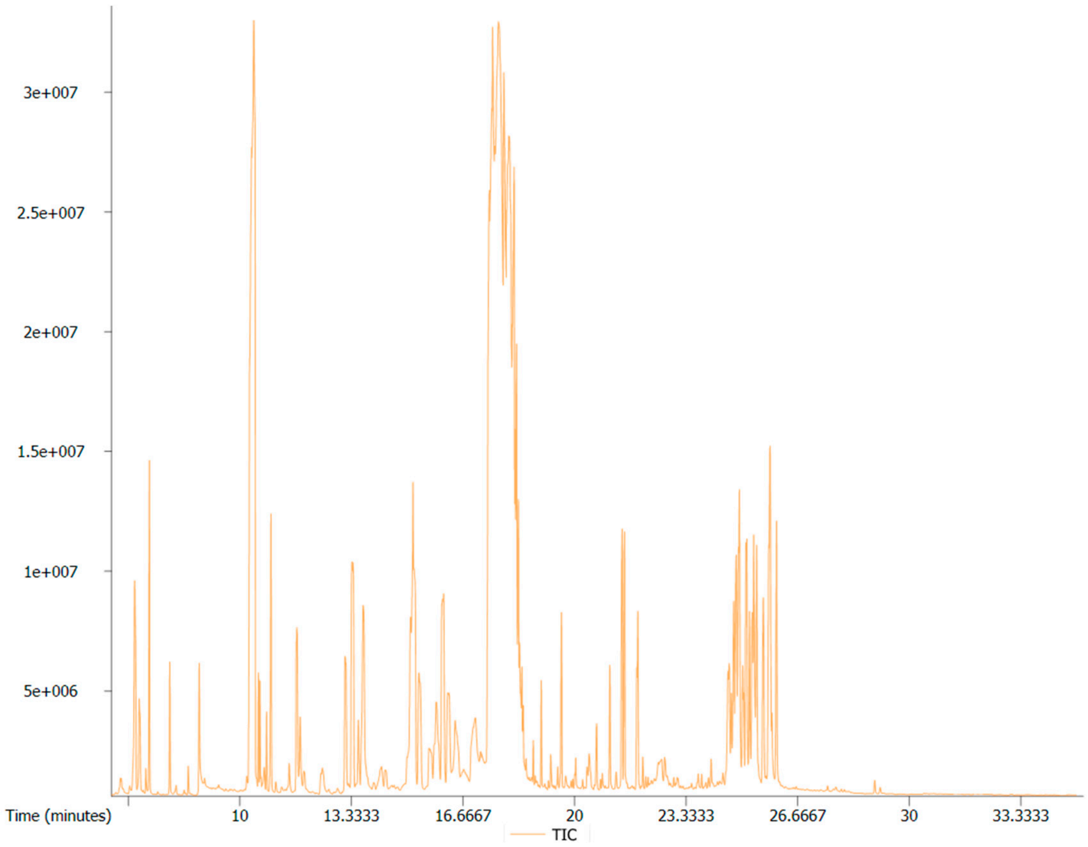

Figure S13. Total ion chromatogram of QC sample on day 21

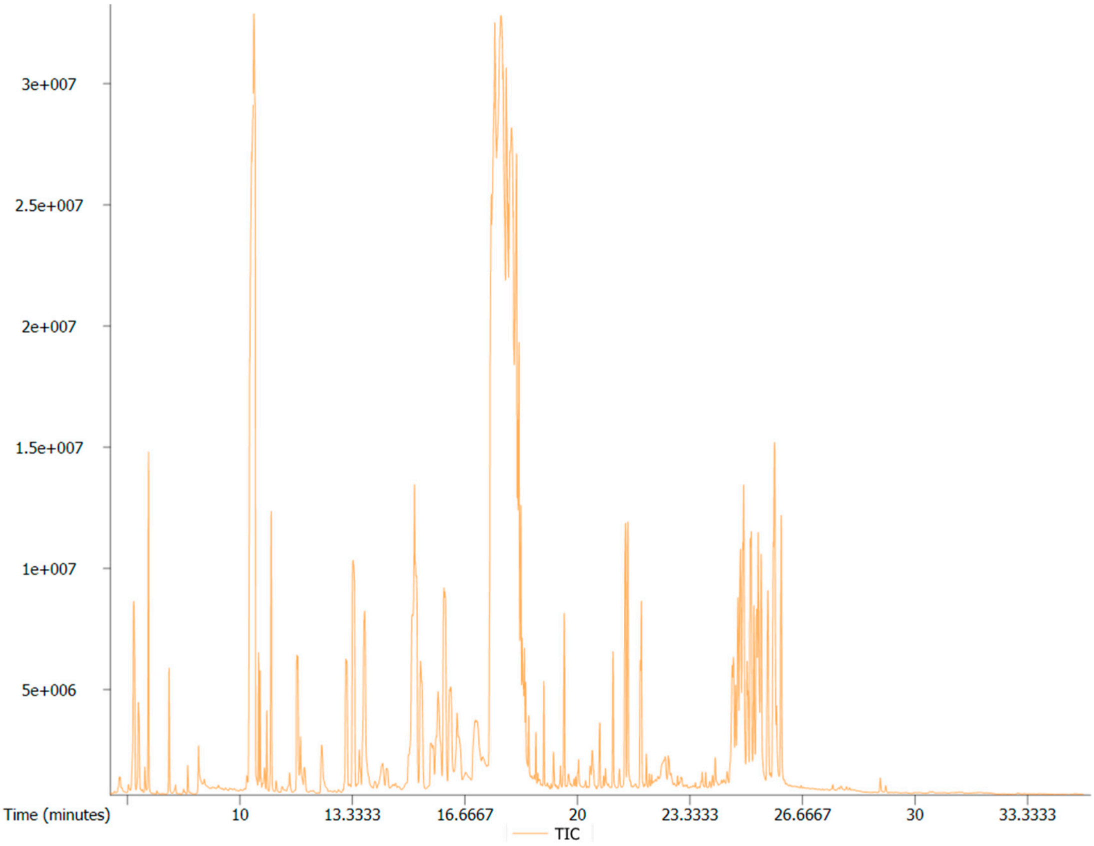

Figure S14. Total ion chromatogram of QC sample on day 21

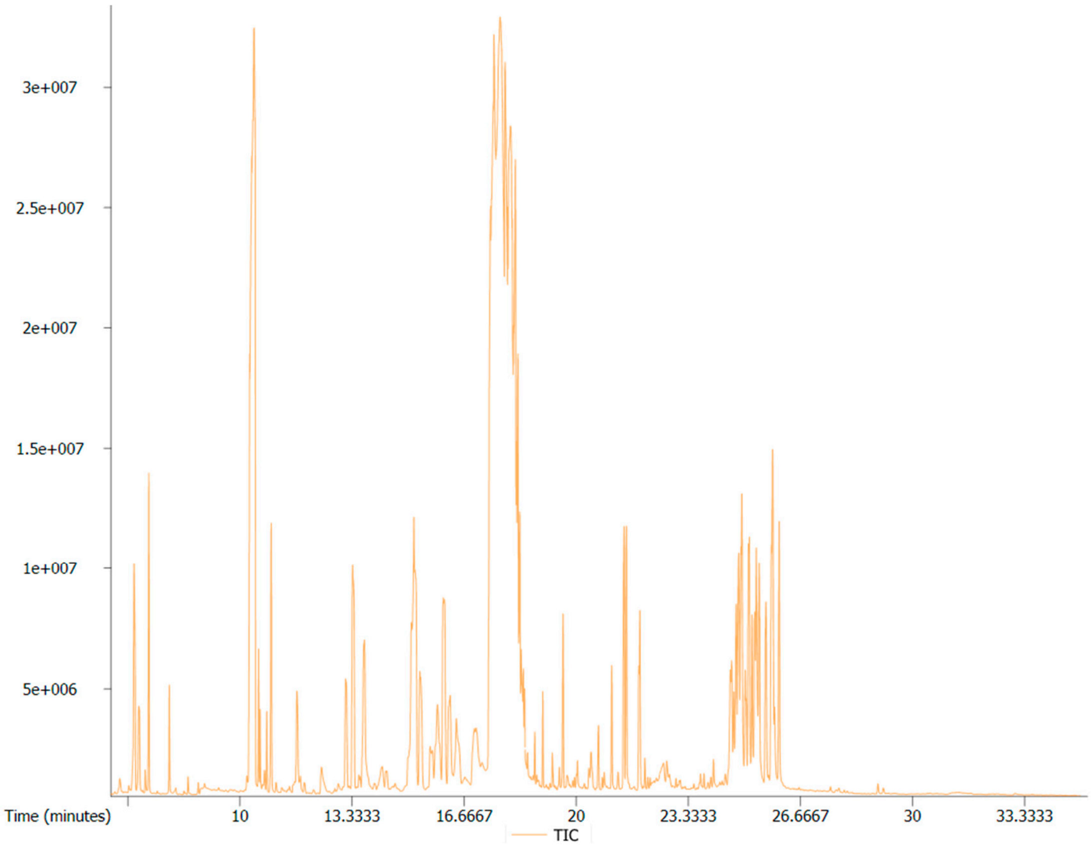

Figure S15. Total ion chromatogram of QC sample on day 21

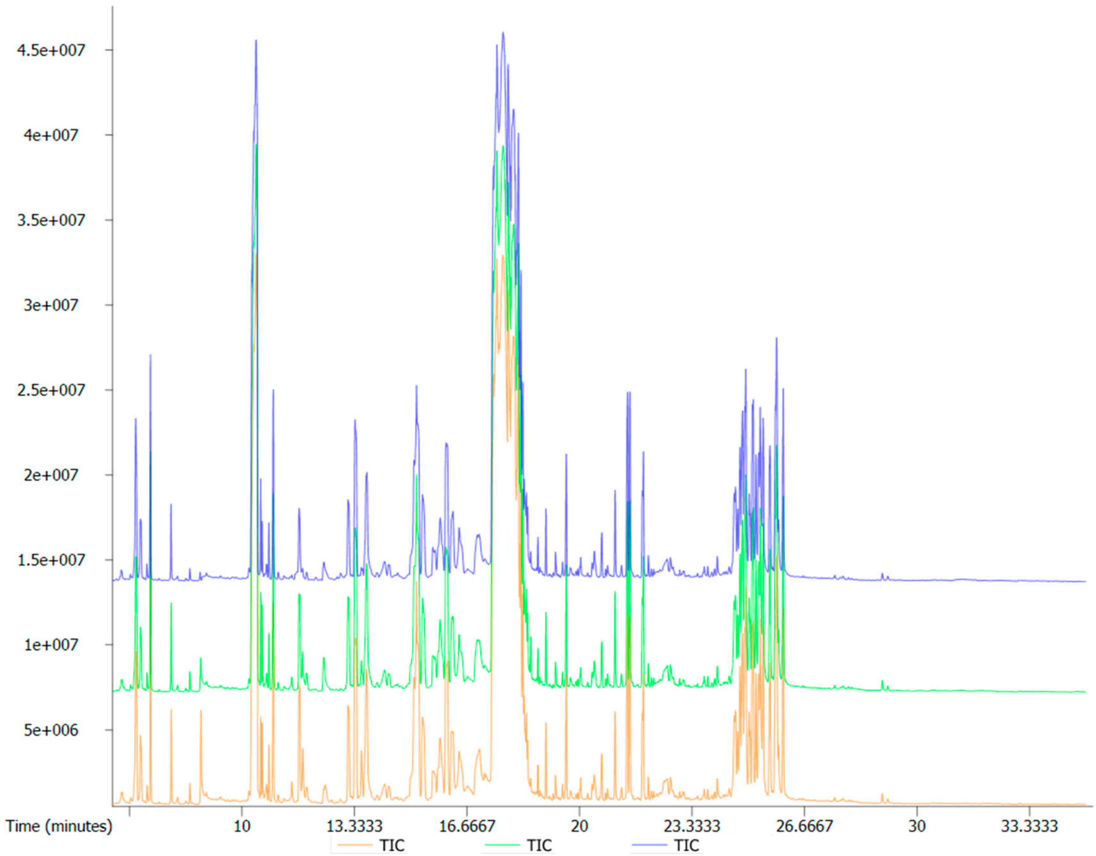

Figure S16. Total ion chromatogram of superimposed QC samples on day 21

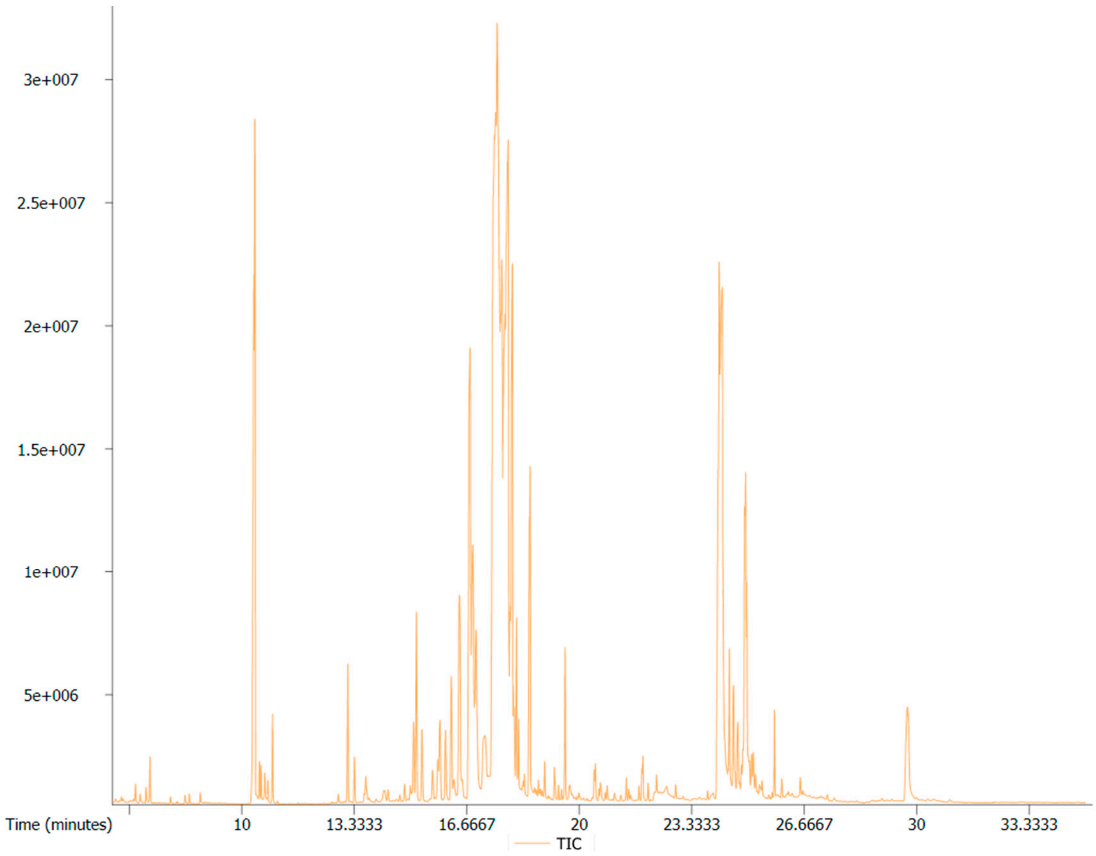

Figure S17. Total ion chromatogram of QC sample on day 30

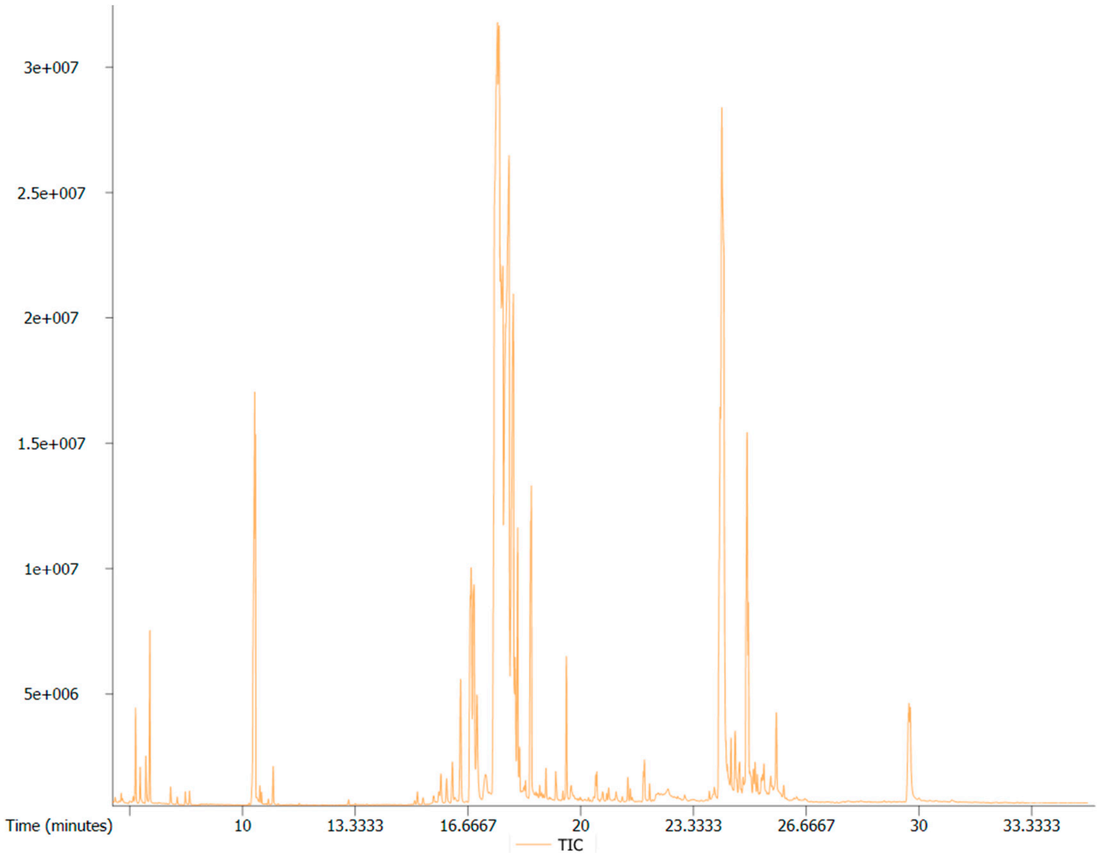

Figure S18. Total ion chromatogram of QC sample on day 30

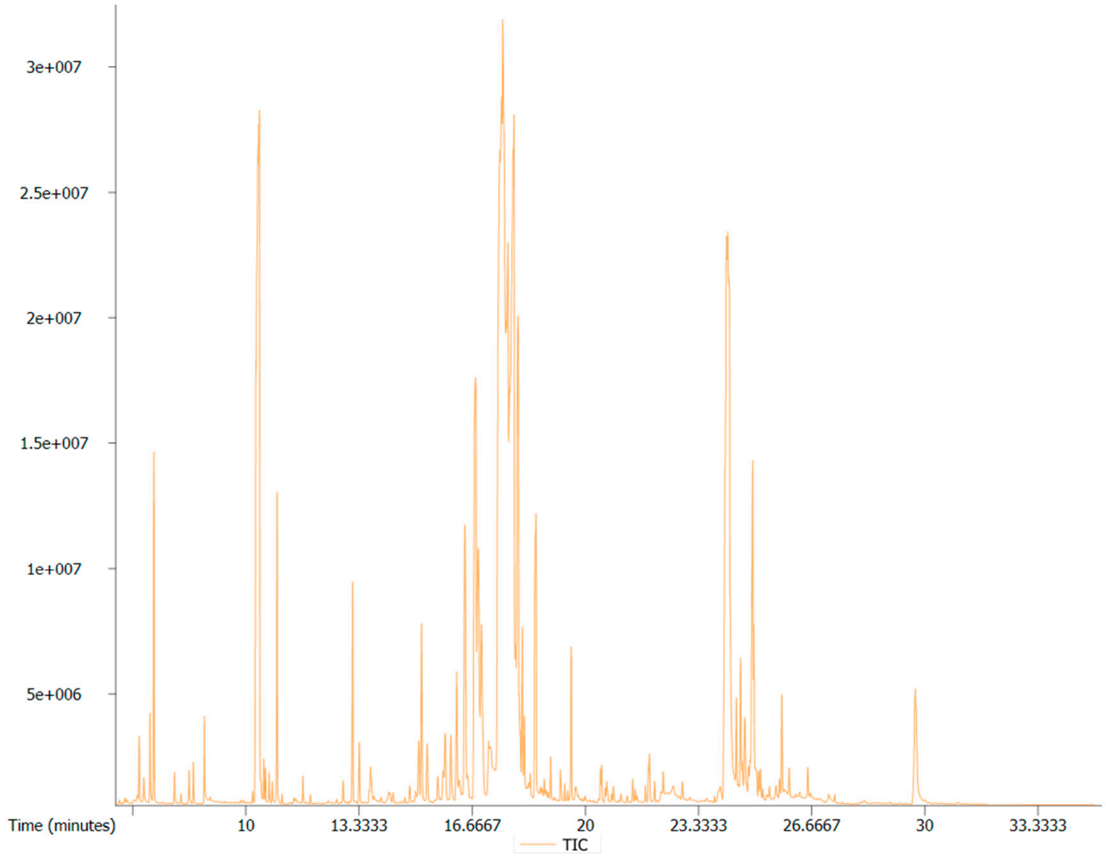

Figure S19. Total ion chromatogram of QC sample on day 30

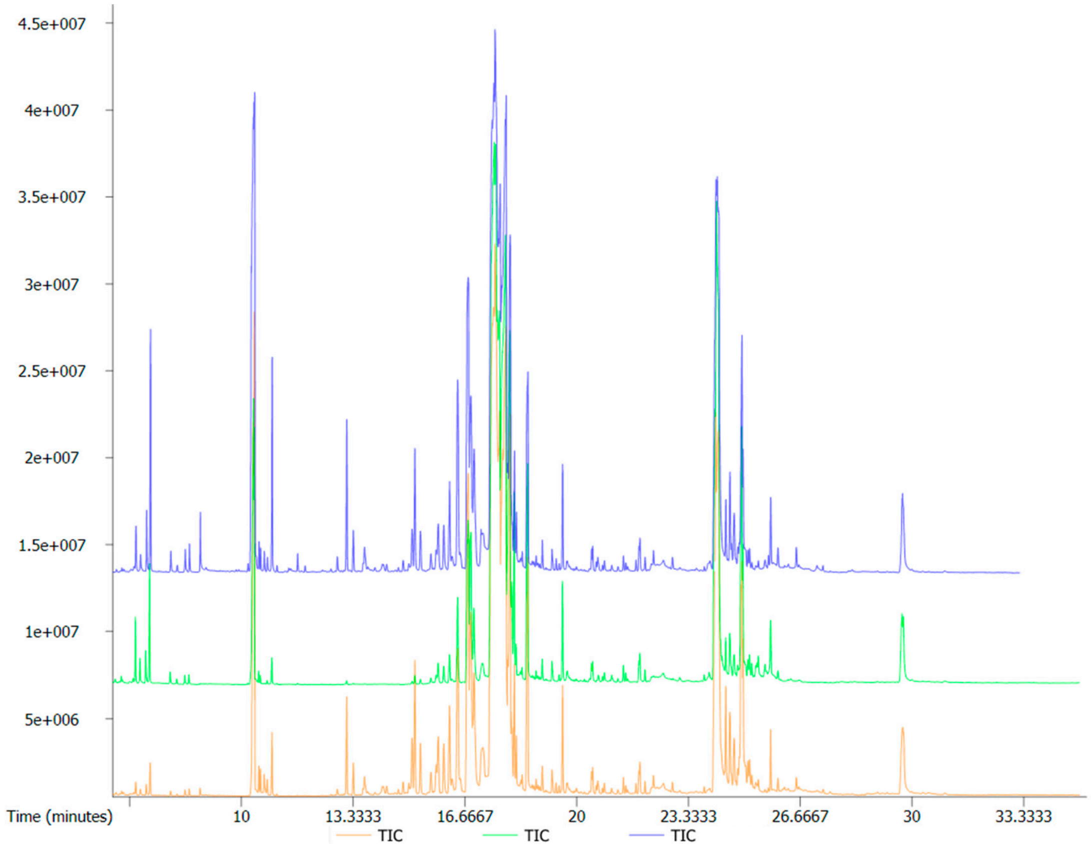

Figure S20. Total ion chromatogram of superimposed QC samples on day 30
