# Supplementary material for: Analysis of Metabolic Components of JUNCAO Wine Based on GC-QTOF-MS
Source: Foods. 2023 Jun 3;12(11):2254. doi: 10.3390/foods12112254 (PMC10252805; doi:10.3390/foods12112254)
Supplement: Supplementary file 1 [file foods-12-02254-s001.zip › foods-2354671-supplementary/foods-2354671-supplementary-sentconversion/Supplementary File/supplementary tables-1.pdf]

Table S1. Differential metabolites in fermented grains from day 0 to day 2

NO.	Log fold chang	VIP	Peak	pvalue
1	-9.6857	1.42	toluenesulfonic acid	1.27E-07
2	-8.6394	1.42	Maleamate	4.33E-06
3	-6.5557	1.36	L-Allothreonine	3.84E-03
4	-5.1459	1.38	3-methylcatechol	2.91E-03
5	-4.8532	1.26	phosphate	1.55E-02
6	-4.6624	1.42	oxoproline	4.39E-05
7	-4.363	1.41	allose	2.69E-04
8	-4.0472	1.42	uridine	1.54E-05
9	-3.9206	1.33	beta-Alanine	9.44E-03
10	-3.8691	1.37	salicin	3.73E-03
11	-3.5925	1.41	4-aminobutyric acid	1.35E-04
12	-3.5056	1.34	Loganin	5.07E-03
13	-3.4595	1.41	salicylic acid	1.27E-04
14	-3.0454	1.38	L-Malic acid	2.04E-03
15	-2.9177	1.31	resorcinol	1.45E-02
16	-2.9165	1.34	Erythrose	7.88E-03
17	-2.9103	1.39	Threonic acid	3.50E-04
18	-2.8353	1.34	3-Aminoisobutyric acid	6.87E-03
19	-2.4031	1.38	Chlorogenic Acid	2.23E-03
20	-2.3789	1.24	D-Glyceric acid	2.00E-02
21	-2.349	1.27	"2,3-Dimethylsuccinic acid"	2.14E-02
22	-2.3167	1.35	N-Acetyl-beta-D-mannosamine	5.90E-03
23	-2.1699	1.27	DL-dihydrosphingosine	2.21E-02
24	-1.9501	1.33	5-Methoxytryptamine	8.64E-03
25	-1.9098	1.24	guanosine	2.56E-02
26	-1.7947	1.40	"Ribonic acid, gamma-lactone"	5.44E-04
27	-1.7539	1.41	D-(glycerol 1-phosphate)	2.28E-04
28	-1.72	1.41	putrescine	1.50E-04
29	-1.5649	1.22	Atropine	3.36E-02
30	-1.5585	1.25	succinic acid	2.95E-02
31	-1.439	1.23	1-Monopalmitin	3.10E-02
32	-1.1796	1.19	"Fructose 2,6-biphosphate degr	4.58E-02

NO.	Log fold chang	VIP	Peak	pvalue
			prod"	
33	-1.1734	1.30	citric acid	1.45E-02
34	-1.0756	1.23	lactose	3.29E-02
35	4.1121	1.27	2-Deoxyuridine	1.37E-02
36	4.1243	1.37	xylitol	3.35E-03
37	4.8881	1.17	beta-Mannosylglycerate	4.28E-02
38	8.1105	1.42	flavin adenine degrad product	1.29E-04

Table S2. Differential metabolites in fermented grains from day 2 to day 10

NO.	Log fold chang	VIP	Peak	pvalue
1	-3.5125	1.38	citric acid	1.25E-02
2	-3.0608	1.41	2-Deoxyuridine	1.00E-02
3	-1.8057	1.35	hydrocortisone	1.71E-02
4	-1.7498	1.37	Aconitic Acid	1.51E-02
5	0.9506	1.25	"2-amino-2-methylpropane-1,3-diol"	4.26E-02
6	1.0215	1.46	2-Deoxyerythritol	4.03E-03
7	1.0662	1.28	2-Ketovaleric acid	3.32E-02
8	1.2062	1.37	D-Glyceric acid	1.45E-02
9	1.3524	1.34	xylose	2.51E-02
10	1.3985	1.29	2-hydroxy-3-isopropylbutanedioic acid	3.85E-02
11	1.4475	1.27	3-Aminoisobutyric acid	3.83E-02
12	1.5166	1.37	malonic acid	1.70E-02
13	1.5184	1.29	4-Hydroxybenzoic acid	3.86E-02
14	1.5676	1.37	Ethanolamine	1.39E-02
15	1.6246	1.47	glycine	2.83E-03
16	1.7009	1.47	maleic acid	2.98E-03
17	1.887	1.35	Gentiobiose	2.00E-02
18	1.9625	1.42	3-Hexenedioic acid	7.91E-03
19	2.0112	1.32	putrescine	2.84E-02
20	2.0274	1.43	4-hydroxybutyrate	5.27E-03
21	2.266	1.28	threonine	4.37E-02
22	2.3349	1.40	Tartronic acid	1.01E-02
23	2.3565	1.34	thymine	2.01E-02

NO.	Log fold chang	VIP	Peak	pvalue
24	2.3579	1.44	pimelic acid	5.23E-03
25	2.524	1.39	3-Hydroxynorvaline	1.03E-02
26	2.7404	1.48	4-aminobutyric acid	1.88E-03
27	2.7785	1.44	3-Hydroxypropionic acid	4.45E-03
28	2.9672	1.50	3-Phenyllactic acid	6.04E-04
29	3.4404	1.51	melibiose	3.25E-04
30	3.6068	1.51	Palatinose	3.75E-04
31	3.6817	1.51	Citramalic acid	1.96E-04
32	3.7229	1.49	Methylmalonic acid	8.55E-04
33	3.8307	1.48	Digitoxose	2.12E-03
34	4.1785	1.42	hydroxylamine	5.64E-03
35	6.9071	1.49	Maleamate	1.25E-02

Table S3. Differential metabolites in fermented grains from day 10 to day 21

NO.	Log fold chang	VIP	Peak	pvalue
1	-2.5041	1.32	"trans-3,5-Dimethoxy-4-hydroxycinnamaldehyde"	9.92E-06
2	-12.474	1.31	1-Kestose	9.74E-05
3	-2.5809	1.31	melibiose	8.12E-05
4	-4.2707	1.32	Galactinol	7.00E-05
5	-0.7545	1.31	3-Hydroxypropionic acid	6.23E-05
6	-5.6318	1.32	naringin	4.59E-05
7	-5.0623	1.32	raffinose	4.26E-06
8	1.2034	1.31	guanine	4.08E-05
9	0.42731	1.09	linolenic acid	4.89E-02
10	-0.42623	1.08	Purine riboside	4.65E-02
11	0.40004	1.10	palmitic acid	4.15E-02
12	-1.903	1.08	Zymosterol	4.02E-02
13	0.76769	1.11	pantothenic acid	3.99E-02
14	7.4077	1.13	beta-Alanine	3.62E-02
15	7.0817	1.09	aspartic acid	3.55E-02
16	-0.45687	1.12	2-hydroxy-3-isopropylbutanedioic acid	3.38E-02
17	1.9896	1.15	Erythrose	3.08E-02
18	-1.0928	1.12	3-Hexenedioic acid	2.96E-02

NO.	Log fold chang	VIP	Peak	pvalue
19	-0.50189	1.12	d-Glucoheptose	2.92E-02
20	0.46068	1.16	cis-sinapinic acid	2.74E-02
21	-0.46098	1.13	4-Hydroxyphenylethanol	2.66E-02
22	-1.5675	1.13	caffeic acid	2.55E-02
23	0.75652	1.16	glutamine	2.39E-02
24	2.2566	1.19	noradrenaline	1.90E-02
25	-1.0464	1.17	Chlorogenic Acid	1.67E-02
26	-0.52684	1.19	Palatinose	1.62E-02
27	-0.6571	1.19	2-Ketovaleric acid	1.55E-02
28	4.2435	1.17	L-Allothreonine	1.54E-02
29	-0.91263	1.18	lactose	1.52E-02
30	-1.0695	1.19	phosphate	1.38E-02
31	-0.64398	1.19	D-Glyceric acid	1.24E-02
32	0.94219	1.22	mucic acid	9.87E-03
33	1.0203	1.23	ribose	9.63E-03
34	0.91339	1.23	xylitol	9.49E-03
35	-2.5112	1.22	4-Hydroxybenzoic acid	9.14E-03
36	-0.38491	1.22	L-Malic acid	7.90E-03
37	-0.65438	1.23	beta-Mannosylglycerate	6.77E-03
38	0.91424	1.23	succinic acid	6.74E-03
39	-0.70499	1.23	oxalic acid	5.34E-03
40	2.8757	1.26	Lyxose	4.42E-03
41	4.7519	1.24	proline	3.79E-03
42	-2.7401	1.27	2-Deoxyuridine	3.32E-03
43	-1.316	1.25	Citramalic acid	3.29E-03
44	-2.4431	1.26	Digitoxose	3.13E-03
45	1.6354	1.28	Aconitic Acid	2.96E-03
46	-0.78068	1.27	"2-amino-2-methylpropane-1,3-diol"	2.32E-03
47	-2.9162	1.27	"Fructose 2,6-biphosphate degr prod"	2.25E-03
48	1.1928	1.28	N-Acetyl-D-galactosamine	2.01E-03
49	-0.75484	1.28	spermidine	1.95E-03
50	-1.8291	1.27	"24,25-dihydrolanosterol"	1.91E-03
51	-3.9782	1.28	prunin degr. Prod.	1.38E-03

NO.	Log fold chang	VIP	Peak	pvalue
52	-3.1948	1.28	Stigmasterol	1.36E-03
53	4.3028	1.29	galactose	1.16E-03
54	1.2744	1.29	alanine	1.15E-03
55	-2.5543	1.28	maleic acid	1.11E-03
56	-2.1028	1.28	pimelic acid	1.00E-03
57	1.2015	1.29	glutathione	9.55E-04
58	-1.5946	1.29	Methylmalonic acid	8.05E-04
59	-1.56	1.29	Gentiobiose	7.63E-04
60	-1.4549	1.30	3-Phenyllactic acid	5.56E-04
61	1.015	1.30	arachidonic acid	4.94E-04
62	-2.8785	1.31	Cetadiol	4.80E-04
63	-1.347	1.29	2-hydroxybutanoic acid	4.31E-04
64	-2.0266	1.31	Neohesperidin	1.56E-04
65	-4.6862	1.31	Melezitose	1.34E-04
66	-4.8391	1.31	sucrose	1.25E-04

Table S4. Differential metabolites in fermented grains from day 21 to day 30

NO.	Log fold chang	VIP	Peak	pvalue
1	-5.4819	1.49	galactose	1.03E-03
2	-3.0943	1.41	Lyxose	9.16E-03
3	-2.6558	1.41	2-hydroxypyridine	7.81E-03
4	-2.1707	1.32	N-Acetyl-beta-D-mannosamine	2.89E-02
5	-2.0513	1.42	xylitol	6.74E-03
6	-1.314	1.33	Erythrose	3.39E-02
7	-0.99743	1.36	succinicacid	7.78E-03
8	-0.96493	1.42	Aconitic Acid	5.66E-03
9	-0.89936	1.35	mucic acid	2.32E-02
10	-0.87542	1.34	glutamine	2.66E-02
11	-0.81607	1.38	lactic acid	1.42E-02
12	-0.79257	1.41	arachidonic acid	6.02E-03
13	-0.67798	1.30	2-Monopalmitin	1.73E-02
14	-0.58885	1.42	oxalic acid	3.93E-03
15	0.41398	1.29	"2-amino-2-methylpropane-1,3-diol"	2.06E-02
16	0.45226	1.21	noradrenaline	4.94E-02

NO.	Log fold chang	VIP	Peak	pvalue
17	0.51368	1.19	fumaric acid	4.84E-02
18	0.54258	1.23	4-hydroxybutyrate	4.86E-02
19	0.56554	1.31	2-Ketovaleric acid	1.64E-02
20	0.62576	1.45	3-Hydroxypropionic acid	1.16E-03
21	0.93168	1.28	D-erythro-sphingosine	3.54E-02
22	1.0335	1.43	Methylmalonic acid	4.54E-03
23	1.0412	1.26	phytosphingosine	3.06E-02
24	1.0561	1.49	Neohesperidin	5.08E-04
25	1.1694	1.31	sucrose	2.01E-02
26	1.2331	1.33	lactulose	1.77E-02
27	1.2543	1.27	Glucose-1-phosphate	1.97E-02
28	1.4193	1.38	arbutin	1.24E-02
29	1.5601	1.32	xylose	3.17E-02
30	1.582	1.37	DL-dihydrosphingosine	1.22E-02
31	1.7288	1.38	Sophorose	8.35E-03
32	1.7456	1.48	"trans-3,5-Dimethoxy-4-hydroxycinnamaldehyde"	7.70E-05
33	1.8097	1.31	maleic acid	1.43E-02
34	1.8221	1.38	cellobiose	8.37E-03
35	1.9626	1.38	Gentiobiose	8.20E-03
36	1.9854	1.40	melibiose	6.87E-03
37	2.0572	1.17	thymidine	4.58E-02
38	2.6098	1.42	Melezitose	6.27E-03
39	2.7849	1.39	Monostearin	5.96E-03
40	2.8718	1.33	trehalose	1.11E-02
41	2.9764	1.41	salicin	8.44E-03
42	3.0009	1.34	androsterone	1.10E-02
43	3.645	1.49	Galactinol	3.97E-04
44	3.6616	1.24	prunin degr. Prod.	3.84E-02
45	5.2817	1.41	sorbitol	5.16E-03
