# Supplementary material for: Analysis of Metabolic Components of JUNCAO Wine Based on GC-QTOF-MS
Source: Foods. 2023 Jun 3;12(11):2254. doi: 10.3390/foods12112254 (PMC10252805; doi:10.3390/foods12112254)
Supplement: Supplementary file 1 [file foods-12-02254-s001.zip › foods-2354671-supplementary/foods-2354671-supplementary-sentconversion/Supplementary File/supplementary tables-2.pdf]

Table S5

Preliminary identification of optimized characteristic peaks of fermented grains during fermentation

Align ID	Retention time	Quantitative ion	Aroma compounds preliminarily annotated	Average similarity
29	7.01917	152	2-Hydroxypyridine	895.00
38	7.28467	117	lactic acid	865.67
41	7.41661	190	glycolic acid	520.57
48	7.89333	116	alanine	975.07
51	8.06	131	hydroxylamine	510.00
54	8.2324	131	2-Hydroxybutanoic acid	677.75
60	8.51583	177	3-Hydroxypropionic acid	870.10
68	8.71917	117	3-Hydroxybutyric acid	808.36
72	8.81417	147	sulfuric acid	838.42
77	8.94101	188	2-Ketovaleric acid	299.38
78	8.94744	130	2-amino-2-methylpropane-1,3-diol	377.80
100	9.76185	159	"2-Butyne-1,4-diol"	321.88
105	9.82993	233	4-Hydroxybutyrate	732.67
109	10.2001	174	Ethanolamine	946.20
112	10.4102	211	phosphate	843.40
113	10.2953	51	glycerol	892.91
114	10.4452	155	2-Deoxyuridine	536.36
120	10.5503	117	2-Deoxyerythritol	922.33
121	10.6039	158	Isoleucine	867.91
124	10.6864	142	proline	844.20
126	10.716	306	maleic acid	459.82
128	10.6701	146	threonine	749.50
129	10.7802	174	glycine	955.20
134	10.9184	55	succinic acid	935.93
138	11.0629	189	D-glyceric acid	944.80
143	11.2547	241	uracil	804.43
147	11.4216	245	fumaric acid	832.73
148	11.4665	204	serine	922.78
153	11.5644	156	Pipecolinic acid	717.13
158	11.7964	219	L-allothreonine	878.69
161	11.9063	131	Tartronic acid	614.67
167	12.0891	255	thymine	798.70
172	12.3983	248	beta-Alanine	664.15
179	12.6511	233	Hydroxynorvaline	374.60
180	12.6748	350	Erythrose	491.71
182	12.7982	174	3-Aminoisobutyric acid	569.57
185	12.9122	232	Aminomalonic acid	554.64
186	12.9189	247	Citramalic acid	805.93

Align ID	Retention time	Quantitative ion	Aroma compounds preliminarily annotated	Average similarity
192	13.1432	233	L-Malic acid	932.27
200	13.3496	156	3-Hexenedioic acid	321.00
203	13.4898	267	salicylic acid	752.43
206	13.5422	232	aspartic acid	922.30
208	13.6426	156	oxoproline	868.86
210	13.6774	174	4-aminobutyric acid	928.60
214	13.7393	292	Maleamate	443.75
223	13.9791	292	Threonic acid	791.57
230	14.1135	120	phenylalanine	816.67
231	14.1914	275	(2R,3S)-2-hydroxy-3-isopropylbutanedioic acid	603.91
232	14.2034	247	2-hydroxy-3-isopropylbutanedioic acid	440.73
234	14.2329	179	4-Hydroxyphenyl ethanol	893.92
235	14.346	142	3-Phenyllactic acid	492.00
240	14.5491	217	pimelic acid	429.25
241	14.5879	204	Digitoxose	450.71
250	14.8223	355	tartaric acid	397.00
256	14.9944	307	Lyxose	882.92
261	15.172	103	xylose	923.00
266	15.3458	103	ribose	898.20
270	15.6332	217	xylitol	854.53
272	15.6022	231	"Ribonic acid, gamma-lactone"	421.67
278	15.7906	307	D-Arabitol	899.33
286	16.0404	204	flavin adenine degrad product	652.93
287	16.0753	174	putrescine	765.93
291	16.2216	229	Aconitic Acid	897.00
294	16.4149	217	Glucose-1-phosphate	813.00
304	16.6724	224	Dehydroshikimic acid	448.00
310	16.9436	273	citric acid	852.77
326	17.9156	160	galactose	819.46
327	17.9489	297	D-Talose	477.44
369	18.4555	333	D-galacturonic acid	912.67
377	18.6886	137	glutamine	463.47
381	18.734	201	pantothenic acid	544.83
386	18.8347	357	succinic acid	300.00
397	19.0977	145	palmitoleic acid	674.50
398	19.1366	333	n-butanol	691.80
399	19.068	353	xanthine	665.43
405	19.2762	117	palmitic acid	937.60
411	19.3867	217	Glucoheptonic acid	652.33
416	19.4873	73	N-acetyl-D-galactosamine	824.47
423	19.5922	305	normal butanol	933.47

Align ID	Retention time	Quantitative ion	Aroma compounds preliminarily annotated	Average similarity
429	19.734	338	ferulic acid	693.62
430	19.7536	319	N-acetyl-beta-D-mannosamine	587.60
435	19.896	174	"trans-3,5-Dimethoxy-4-hydroxycinnam aldehyde"	380.86
437	19.9616	57	cis-Phytol	309.50
438	19.996	319	d-Glucoheptose	659.40
441	20.0379	251	Flavanone	368.50
442	20.0448	352	guanine	691.45
444	20.0933	219	caffeic acid	571.08
462	20.4075	143	4-normal butanol	733.50
466	20.4342	79	glutathione	344.38
477	20.6548	204	beta-Mannosylglycerate	789.57
478	20.69	227	isoamylol	820.30
481	20.7718	67	linoleic acid	890.00
482	20.8245	117	oleic acid	851.00
483	20.8329	79	linolenic acid	853.40
492	21.0433	117	stearic acid	829.14
494	21.0626	84	spermidine	341.14
515	21.5539	56	Atropine	302.29
533	22.0375	204	Purine riboside	651.00
537	22.1412	325	arachidonic acid	295.92
556	22.4915	204	D-erythro-sphingosine	565.40
56	8.32622	147	oxalic acid	933.40
564	22.7332	224	uridine	745.00
566	22.7677	259	cytidine-monophosphate degr prod	773.80
574	23.0115	204	DL-dihydrosphingosine	593.21
577	23.0707	446	Neohesperidin	501.00
584	23.2525	73	salicin	609.38
597	23.5412	129	2-Monopalmitin	425.83
599	23.583	297	kyotorphin	407.18
605	23.8021	371	1-Monopalmitin	924.07
615	24.0961	361	sucrose	901.83
638	24.5080	314	lactose	293.50
643	24.6241	172	lactulose	858.93
646	24.7502	261	cellobiose	889.75
671	25.2239	319	Monostearin	733.67
672	25.2445	461	Cetadiol	171.00
678	25.3483	319	Sophorose	835.55
680	25.4254	160	Gentiobiose	947.27
685	25.6199	361	Palatinose	895.85
689	25.7983	361	Isomaltose	875.08
696	26.0026	361	melibiose	799.07

Align ID	Retention time	Quantitative ion	Aroma compounds preliminarily annotated	Average similarity
704	26.1982	297	Loganin	212.92
707	26.2972	204	Galactinol	672.00
717	26.6258	119	hydrocortisone	459.29
734	27.3683	219	Chlorogenic acid	368.75
760	28.5191	297	Aldosterone	295.00
771	29.3395	280	Stigmasterol	280.75
772	29.4128	361	naringin	426.44
773	29.4604	129	"24,25-Dihydrolanosterol"	469.73
774	29.5665	361	raffinose	688.08
776	29.7231	217	1-Kestose	845.40
780	30.4977	361	Melezitose	678.86
82	9.03772	241	Methyl phosphate	794.13
93	9.3672	233	malonic acid	842.07
95	9.48673	144	valine	885.67
96	9.56715	160	Methylmalonic acid	424.17
