# Supplementary material for: Analysis of Metabolic Components of JUNCAO Wine Based on GC-QTOF-MS
Source: Foods. 2023 Jun 3;12(11):2254. doi: 10.3390/foods12112254 (PMC10252805; doi:10.3390/foods12112254)
Supplement: Supplementary file 1 [file foods-12-02254-s001.zip › foods-2354671-supplementary/foods-2354671-supplementary-sentconversion/Supplementary File/supplementary text.pdf]

Variation of the relative contents of different metabolites during fermentation.

The relative content of 40 different metabolites was used to plot the heat map for intuitively discovering the changes in differential metabolites during fermentation (Figure 5). In the fermentation process, according to the importance of variables, the different compounds screened can be mainly divided into four categories: organic acids, amino acids, sugars, sugar alcohols, and other compounds.

We found that 21 organic acids were differential metabolites. From 0 to 2 days of fermentation, citric acid and 4-aminobutyric acid dominate, followed by the contents of L-malic acid, oxalic acid and phosphoric acid. Aconite acid and glucose-1-phosphate were also found to be high at day 0 of fermentation, indicating that the fermenting feedstock contained these organic acids. Lactic acid is produced from pyruvate by the action of lactate dehydrogenase. It reaches its maximum value on the 10th day of fermentation, after which it gradually decreases, probably due to the co-fermentation of lactic acid and yeast and the conversion into ethyl lactate in the alcohol reaction. Citric acid, glucose-1-phosphate, and L-malic acid are metabolic intermediates in glycogen production and decomposition, and play a significant role in the forming of wine flavor. The citric acid content was the highest on day 0 of fermentation, which may be related to the addition of a small amount of citronella in the fermenting raw material. The acetic acid content was highest at day 21 of fermentation, while the 4-aminobutyric acid content decreased sharply from day 0 to day 2 of fermentation, and then increased rapidly from day 2 to 10. After that, it maintained a relatively steady trend, which had a significant impact on the flavor of the wine. As the fermentation time is extended, the oxalic acid content gradually decreases, and the oxalic acid content is shallower at the end of fermentation, which may be due to the involvement of the phospholipid glycolysis pathway and the phosphorylation of ADP in the TCA cycle. The linolenic acid content reached its maximum at 2 days of fermentation and the arachidonic acid at 21 days. The amount of unsaturated fatty acids first increases and then decreases.

Fluctuating changes in sugars were observed throughout fermentation. The high galactose content at day 0 indicates that the sugars in the raw material are mainly galactose. On the second day of fermentation, the amount of galactose and 1-saccharose increased, and 1-kestose being the second most dominant carbohydrate. The rapid increase of 1-kestose content on day 2 of fermentation may be attributed to the combination of sucrose with fructose-group through the β-2-1 bond. The proportion of galactose decreased significantly on the day 10 of fermentation, suggesting that galactose was converted to 1-glucose phosphate under the catalysis of β-lactase, galactose-1-uridine phosphotransferase, and uridine diphosphate galactose epiisomerase during this period. On the day 10 of fermentation, the content of xylose increased significantly increasesand becames the absolute dominant sugar, followed by the higher levels of 1-kestose. The analysis of the

large increase in xylose content during the early stages of fermentation may be attributed to the fact that the fermenting raw material contains abundant hemicellulose, which is mostly pentose with a small amount of polyhexose. As the fermentation time lengthens and the temperature of the fermenter rises, the pentose will partially decompose into xylose and aracose, and a large amount of xylose will accumulate during the fermentation stage. On day 21 of fermentation, galactose, xylose and ribose were dominant, with galactose increasing significantly during this period. It has been analyzed that the reason for the decrease in the proportion of xylose decreased during this fermentation period is that the yeast converts xylose to xylitol by xylose reductase and reduced it to L-lactic acid by the catalytic action of glyceraldehyde 6-phosphoglucose 3-phosphate and L-lactate dehydrogenase. At the same time, xylose is metabolized to galactose by lactic acid bacteria via the phosphoketolase pathway. The amount of xylose at the end of fermentation on day 30 was three times higher than on day 21, probably due to the difficulty of using xylose by the large number of microorganisms at the end of fermentation. Second, the ribose content is higher. The reason may be that sucrose began to be metabolized by microorganisms after 10 day or was hydrolyzed by microbial hydrolytic enzymes to produce fructose and glucose, leading to a rapid decline in its content. The reason may be that sucrose begins to be metabolized by microorganisms after 10 days or is hydrolyzed by microbial hydrolytic enzymes to produce fructose and glucose, leading to a rapid decline in its content. Erythritose is a monosaccharide that is not used by yeast, so its content changes little during fermentation. The melibiose content is shallow in the early stage of fermentation. However, it gradually increased with the extension of fermentation time and reached the highest level on the day 10 of fermentation, during which the galactose dropped dramatically, suggesting that part of the galactose was converted into melibiose under the action of yeast. The raffinose content during fermentation increases slightly at first, reaching a maximum on day 2, before falling to very low levels. The raffinose content of may be involved in the conversion of galactose metabolism pathway to sucrose by the galactosidase pathway during the middle and late stage of fermentation.

In this study, hydroxyproline levers were the highest at day 0 of fermentation, followed by alanine and aspartic acid. The high proportion of hydroxyproline on day 0 of fermentation may be due to the abundance of hydroxyproline in the brewing material. On the day 2 of fermentation, the hydroxyproline content drops dramatically and the aspartic acid content increases. The content of hydroxyproline and alanine increased gradually after 10 day of fermentation. The amount of aspartic acid decreases rapidly on day 10 of fermentation, then increases significantly on day 21, and then decreases again on day 30 of fermentation. Hydroxyproline, aspartic acid, and alanine dominate the amino acids throughout the fermentation. During fermentation, the proline content is highest on the second day of fermentation and lowest on the 10th day, then gradually

increases. Glutamine is a slightly sweet non-essential amino acid that provides the body with an essential source of nitrogen. It is involved in the synthesis of glutathione, which is obtained in the body through the conversion of glucose, and through the synthesis of glutamic acid, valine and isoleucine. The contents of glutamine, threonine, and β-alanine were low in the fermentation process.

In addition, purine and 2-hydroxypyridine nucleoside were the highest, followed by salicin, thymine and loganin. The glutathione content varied significantly different from 10 days to 21 days of fermentation.
